# Core-valence double ionization of carbon suboxide

**DOI:** 10.1038/s41598-025-01057-4

**Published:** 2025-05-06

**Authors:** Emelie Olsson, Lucas M. Cornetta, Veronica Daver Ideböhn, Måns Wallner, Marco Parriani, Richard J. Squibb, Gunnar Öhrwall, Stefano Falcinelli, Leif Karlsson, John H. D. Eland, Hans Ågren, Raimund Feifel

**Affiliations:** 1 Department of Physics, University of Gothenburg, Origovägen 6B, 412 58 Gothenburg, Sweden; 2 Instituto de Física da Universidade de São Paulo, R. do Matão, 1371, São Paulo, 05508-090 Brazil; 3 Department of Civil and Environmental Engineering, University of Perugia, Via G. Duranti 93, 06125 Perugia, Italy; 4 MAX IV Laboratory, 118, 221 00 Lund, Sweden; 5 Department of Physics and Astronomy, Uppsala University, 516, 751 20 Uppsala, Sweden; 6 Physical and Theoretical Chemistry Laboratory, Department of Chemistry, Oxford University, South Parks Road, Oxford, OX1 3QZ UK; 7 Faculty of Chemistry, Wroclaw University of Science and Technology, Wyb. Wyspianskiego 27, 50370 Wrocław, Poland

**Keywords:** Atomic and molecular interactions with photons, Electronic structure of atoms and molecules

## Abstract

We have measured the core-valence double ionization spectra of carbon suboxide above both the O 1s and C 1s edges. Following several core-valence cases known in the literature, to begin with the spectra are compared with the well-known single ionization valence photoelectron spectrum of this system, from which they surprisingly differ quite strongly. This motivates a comparison to electronic structure calculations carried out within the sudden approximation where the overlap between the core and valence orbital is included, while still assuming decoupling of the core and valence ionization events. The substantially improved agreement indicates that this more complex description is needed to model the core-valence double ionization process adequately in the present case. Accordingly, assignments of spectral features are made by comparison of our experimental and numerical spectra. Auger decay following C 1s hole formation at the chemically distinct central and outer C atoms shows strong selectivity in the final multiply charged states produced, for initial core-valence ionization, being consistent with the Auger decay from single core ionization. Comparison to calculations allows for the identification of the initial core ionization site for the Auger decay following core-valence ionization.

## Introduction

Carbon suboxide, C$_3$O$_2$ or O=C=C=C=O, is a highly symmetric and unstable molecule which is not only of great fundamental interest, but is also used in the preparation of malonates (i.e. the ionized forms of malonic acid CH$_2$(COOH)$_2$) and as an auxiliary to improve the dye affinity of furs. Moreover, in chemical synthesis, carbon suboxide is a 1,3-dipole, reacting with alkenes to make 1,3-cyclopentadiones. Because it is so unstable, it is a reagent of last resort^1^.

Considerable effort has already been put into determining its electronic and geometric structure in different charge states. For a long time its true geometric structure in neutral form was debated as linear, bent or quasi-linear, until the early 1990’s when Vander Auwera et al.^2^ showed by means of infrared spectroscopy that C$_3$O$_2$ is quasi-linear. C$_3$O$_2$ has an unusually large negative charge on the central carbon atom, which gives it a tendency to bend at the central atom.

The single ionization valence photoelectron spectrum of C$_3$O$_2$ was first measured by Baker and Turner in 1968^3^, and later by Rabalais et al.^4^ with higher resolution, revealing its vibrational structure. Around the same time, its electronic structure was also investigated by Gelius et al.^5^, who measured not only the valence region, but also the inner shell regions of the C$_3$O$_2$ photoelectron spectrum using ESCA, where strong shake-up contributions upon core ionization were found. They also determined the chemical shift between the central and outer C 1s edges in gaseous C$_3$O$_2$ to 3.4 eV. Subsequently, the Auger electron spectra at both the C 1s and O 1s edges were reported by Karlsson et al.^6^, and showed large differences in atomic orbital populations of different states of the molecular doubly-charged ion depending on the identity of the atom where core ionization occurred. The differences accorded with the expectation that a low electron population at an atom should result in low Auger intensity for ejection of an electron from the corresponding orbital. These investigations were aided by calculations from which the molecular orbital order in C$_3$O$_2$ outside the inner shells was determined as:

$$\begin{aligned} {... 4\sigma _\text{g}^2 3\sigma _\text{u}^2 5\sigma _\text{g}^2 4\sigma _\text{u}^2 6\sigma _\text{g}^2 5\sigma _\text{u}^2 1\pi _\text{u}^4 1\pi _\text{g}^4 2\pi _\text{u}^4} \end{aligned}$$

In this work, we extend the present knowledge on the electronic structure of carbon suboxide by focussing on the core-valence double ionization of this species which we measured above both the C 1s and O 1s edges. Previous investigations of related molecular systems have shown that core-valence spectra can be expected to resemble the valence single ionization spectra of the same molecule if the core orbital has essentially no overlap with the valence shells (e.g. Ref.^7^), and may even reveal strong molecular symmetry-breaking effects as very recently reported for the highly symmetric allene molecule^8^. In addition to that, Auger electron spectra from both the core-valence double and core single ionization processes are presented and compared, utilizing our unique capability to select Auger spectra in coincidence with individual core-hole components.

## Results and discussion

As described in more detail in the Methods section, the core-valence double ionization spectra were measured using multi-electron coincidence detection and setting the photon energy sufficiently high above the carbon and oxygen 1s inner shells, respectively, to enable a valence electron to be emitted together with the chosen inner shell electron. The available excess energy will be shared between the core and the valence electron, and to determine the double ionization energy, the sum of the two electron energies is subtracted from the photon energy. Since there is an initial inner shell hole and the atoms involved are relatively light, the probability for Auger decay to take place is very high. For single Auger decay, which is the simplest and most probable case, the resulting ion will be triply charged. Thus, in analysing triple coincidence events, energy selection on the secondary Auger electrons, which are easily identified because their kinetic energies are independent of the photon energy, helps to distinguish the sought-for events of core-valence electron ionization from concurrent events such as double Auger decay.

**Fig. 1:**
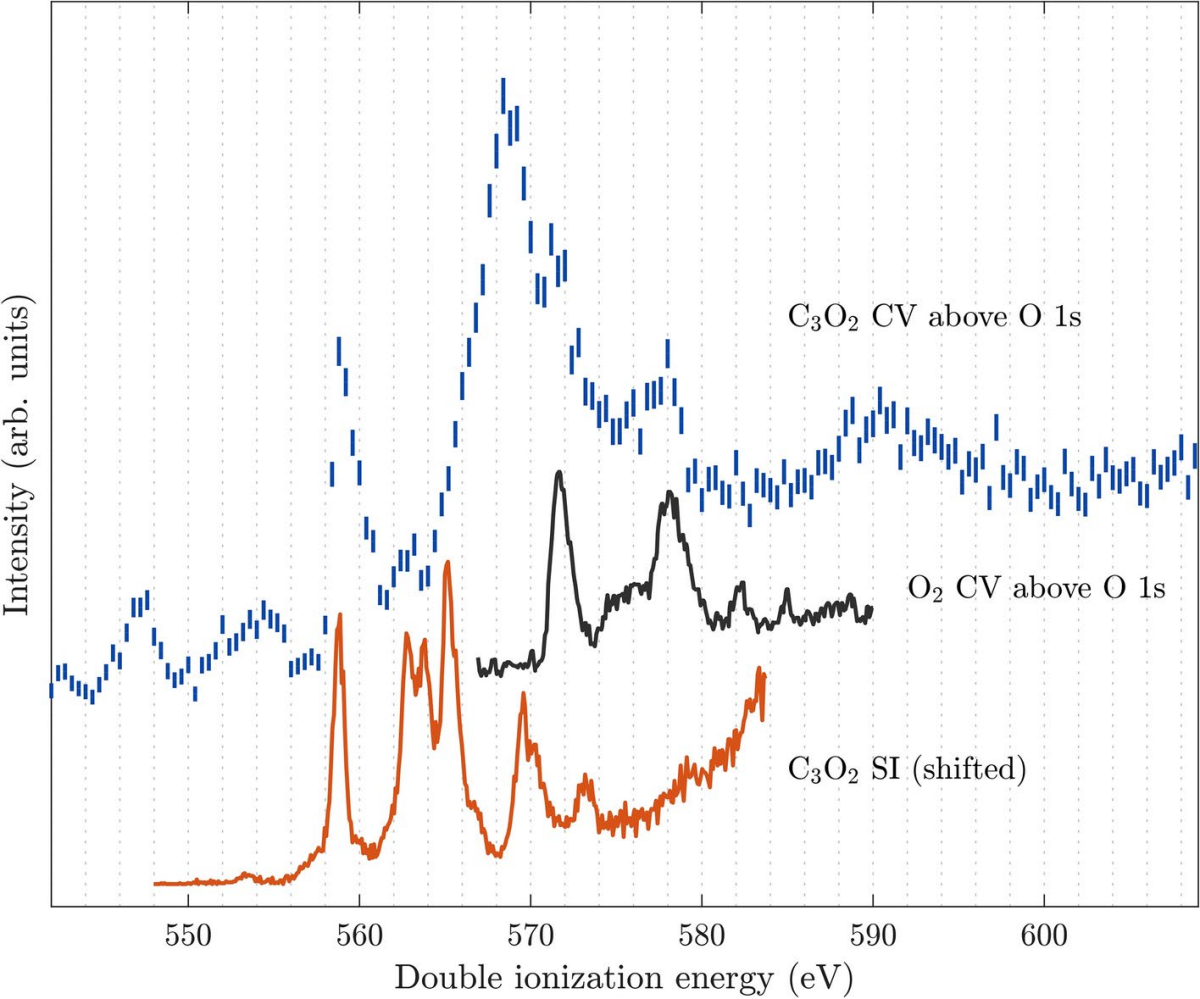
Core-valence (CV) ionization spectrum of C$_3$O$_2$ above the O 1s edge, measured at the photon energy of 618 eV (top spectrum). For comparison, the single ionization (SI) valence photoelectron spectrum for C$_3$O$_2$ obtained at 40.8 eV (bottom spectrum), shifted to align with the first peak of the core-valence spectrum, is included. The core-valence spectrum of O$_2$ taken from the work of Andersson et al.^7^ is also displayed in the middle part, because O$_2$ might be present as an impurity.

**Fig. 2:**
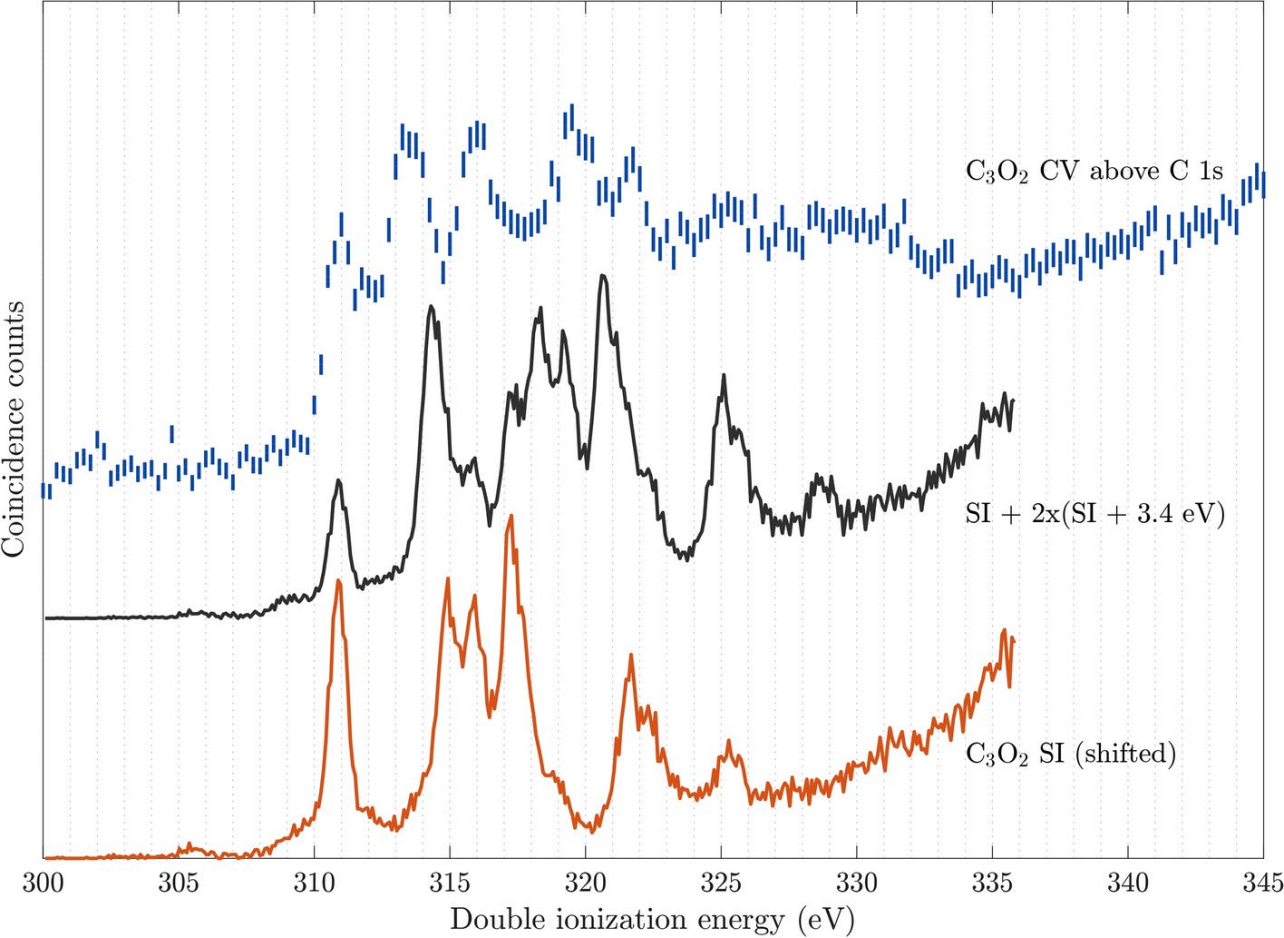
Core-valence (CV) double ionization spectrum of C$_3$O$_2$ above the C 1s edge, measured at the photon energy of 374 eV (top part). For comparison, the lowest curve is the single ionization (SI) valence photoelectron spectrum of C$_3$O$_2$ obtained at 40.8 eV. The central curve shows the sum of two single ionization valence photoelectron spectra, shifted relative to each other by the C 1s core hole difference of 3.4 eV and added together with relative intensities near 2:1, reflecting the intensities of lines from the outer and central carbon atoms. The energy scales of the both comparison spectra were shifted to align with the first peak of the core-valence spectrum.

In the top part of Fig. 1, the core-valence electron spectrum of C$_3$O$_2$ above the O1s edge is displayed in blue, with error bars reflecting the statistical uncertainty. The spectrum shows a first peak at about 558.8 eV, and a stronger second peak, with maximum intensity at 568.4 eV. The resolution for the double ionization energy spectrum is about 1 eV, estimated from the kinetic energy of the electrons in the spectrum. For comparison, the lower part of Fig. 1, shows in orange the single ionization valence photoelectron spectrum of C$_3$O$_2$ measured with the same spectrometer at a photon energy of 40.81 eV, shifted to align the first peaks of the single ionization and core-valence spectra.

The electronic structure of core-ionized C$_3$O$_2$ was investigated in the past by Gelius et al.^5^, where the O 1s binding energy was determined to be 539.7 ± 0.1 eV. Their O 1s core level photoelectron spectrum also showed shake-up features located at about 548 eV and 555 eV (i.e. about 8 eV and 15 eV above the O 1s edge). This shake-up contribution could possibly be the origin of the two structures located below the first main core-valence peak in Fig. 1, if the shake-up electron is detected in coincidence with another, randomly scattered electron. Auger decay of the core hole is expected in both the case of the single ionization shake-up process and the case of the core-valence double ionization process. The Auger spectra from the single core ionization and the Auger spectra from core-valence ionization are found to be so similar within the energy resolution available, that no extra selection of core-valence events based on characteristic Auger electron energies is possible. Shake-up should not normally contribute to the triple coincidence events from which the core-valence spectrum is derived.

In the single core ionization spectrum of C$_3$O$_2$, also extracted from the same data set measured at the photon energy of 618 eV, the O 1s photoelectron lines of O$_2$ are visible at binding energies of 543.4 eV and 544.5 eV^9^ with a relative intensity compared to the C$_3$O$_2$ photoelectron line of about 10 %. This O$_2$ probably originates from air contamination, since we do not expect any O$_2$ directly from the synthesis (see Methods section). We also detected the photoelectron lines of N$_2$ (N 1s at 409.9 eV), which further supports this explanation. The contaminating O$_2$ signals cannot be removed selectively from the spectrum, because the O 1s Auger electron energies of the two molecules are too similar. However, in Fig. 1, the middle spectrum in black is the known core-valence double ionization spectrum of O$_2$ taken from Andersson et al.^7^ which has its first peak at 571.6 eV, showing that at least the first two peaks of the O 1s core-valence spectrum of C$_3$O$_2$ are not affected by the presence of O$_2$.

**Fig. 3:**
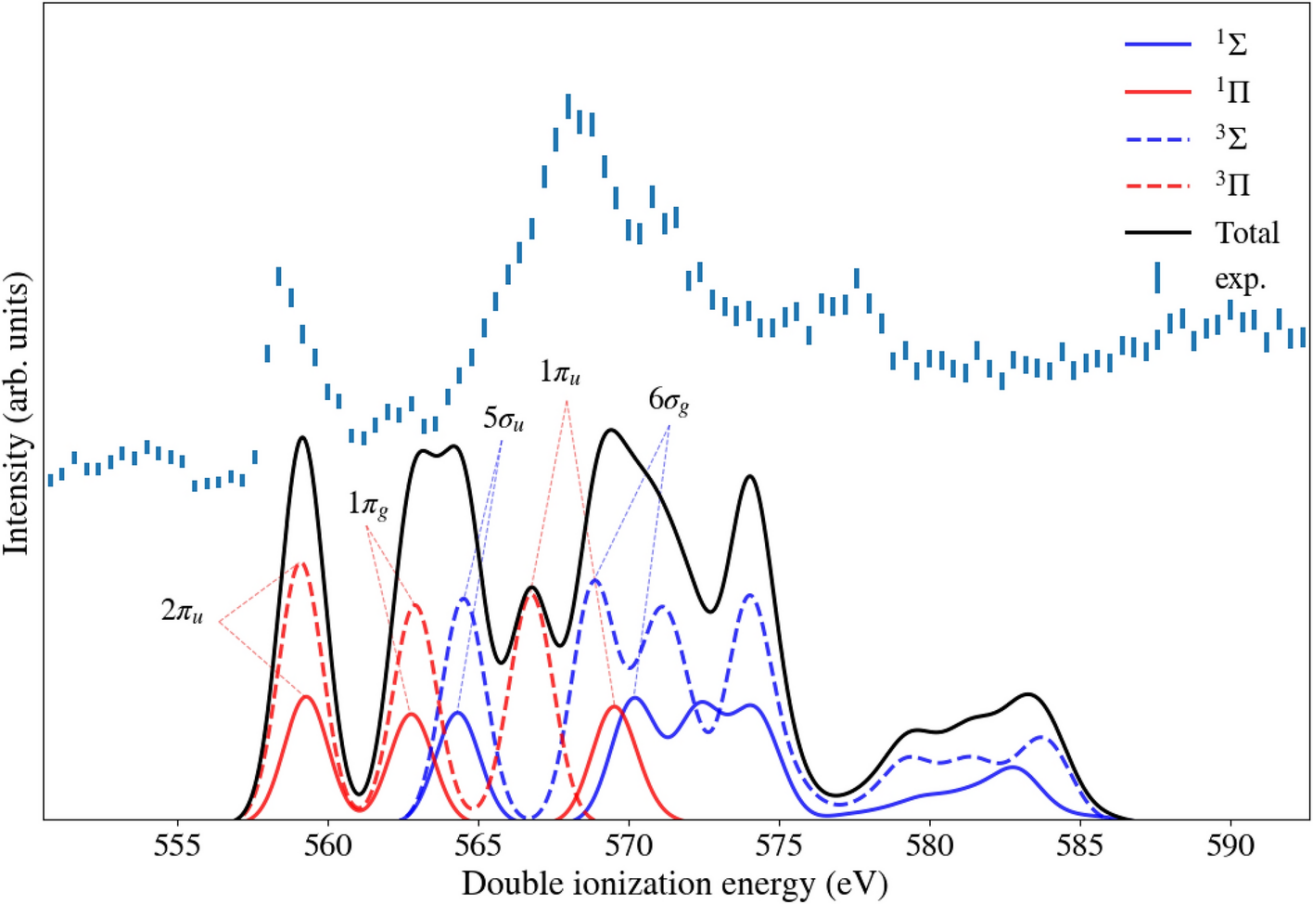
Core-valence ionization spectrum of C$_3$O$_2$ above the O 1s edge, measured at a photon energy of 618 eV (top spectrum), and for comparison, a core-valence spectrum from RASCF/RASPT2 calculations (bottom spectrum).

**Fig. 4:**
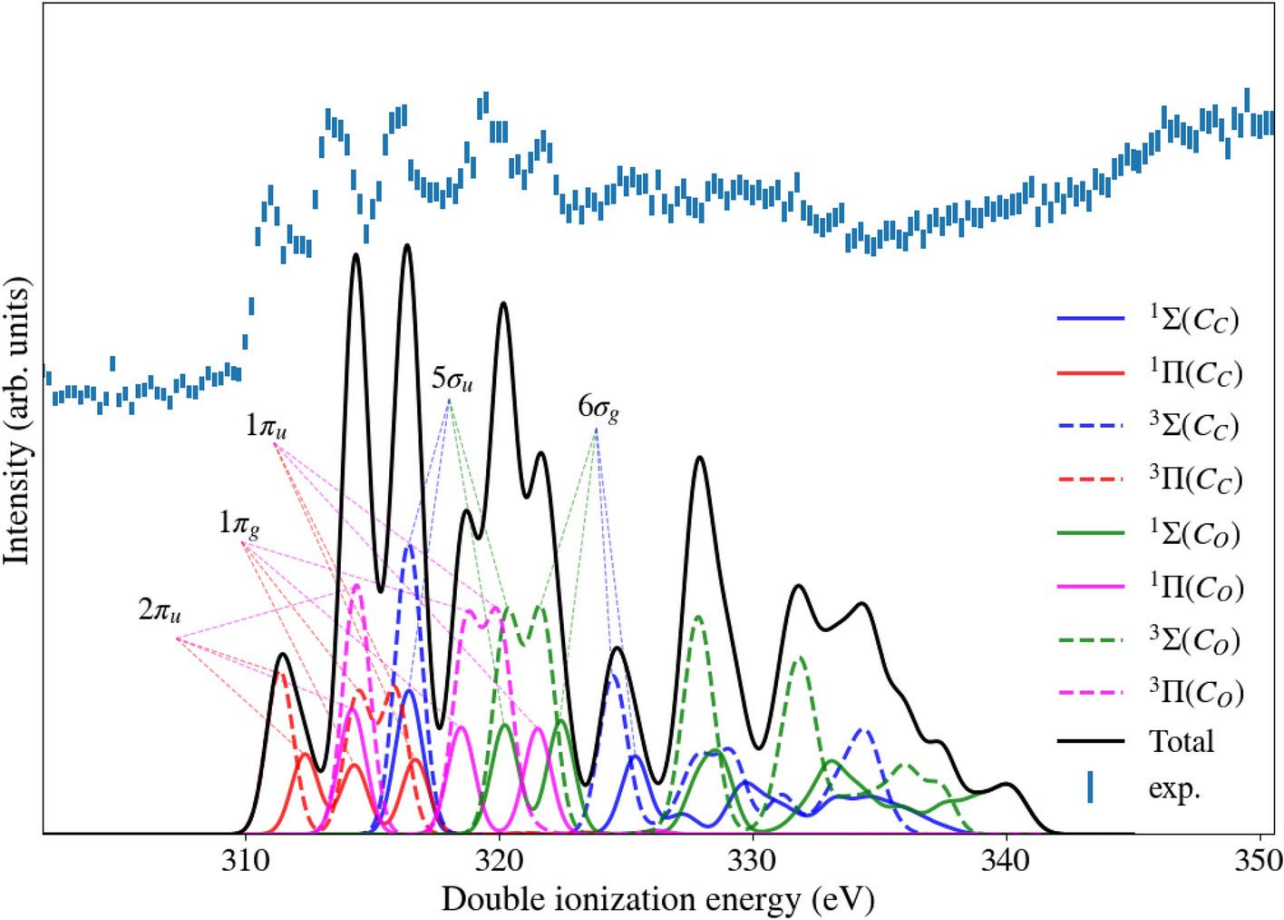
Core-valence ionization spectrum of C$_3$O$_2$ above the C 1s edge, measured at a photon energy of 374 eV (top spectrum), with the core-valence spectrum from RASCF/RASPT2 calculations for comparison (bottom spectrum).

The core-valence ionization spectrum of C$_3$O$_2$ above the C 1s edges measured at a photon energy of 374 eV, i.e. about 70 eV above the C 1s edges, is presented in Fig. 2. It contains five distinct features with rather comparable intensities at about 311.0, 313.2, 316.0, 319.5 and 321.8 eV. The resolution of the double ionization spectrum, at these electron kinetic energies, is again about 1 eV.

According to the work of Gelius et al.^5^, the chemically different carbon atoms of C$_3$O$_2$ have 1s binding energies of 294.9 ± 0.1 eV and 291.5 ± 0.1 eV for the outer and central sites, respectively. Our own measurements (Supplementary Table S1) are in excellent agreement with these values. The intensity ratio between the photoelectron lines for the outer and central carbon atoms is 1.8 according to Gelius et al.^5^, when measured at a much higher photon energy (MgK$_\alpha$ at 1253.6 eV). From our data, the ratio is somewhat above 2, possibly suggesting that there could be an additional contribution to the higher binding energy peak if one assumes the relative ionization cross-sections to be the same as in the Mg K$_\alpha$ excited study of Gelius et al.^5^. The most likely cause for such a discrepancy could be acetic acid originating from the synthesis, CH$_3$COOH, with an expected C 1s binding energy 295.4 eV^10^. Even if acetic acid could be identified based on the photoelectron lines, it is not possible to estimate its contribution to the core-valence spectra from the intensities of these features, since the relative cross sections for the two processes in acetic acid and C$_3$O$_2$ might very well be different. However, the relative amount of acetic acid entering the spectrometer generally decreases over time as observed in the related electron-ion single ionization measurements carried out in our laboratory in Gothenburg. When core-valence spectra taken with a fresh sample, where acetic acid is expected to contribute most, are compared with core-valence spectra taken with the same sample after some hours, no appreciable difference can be found. This indicates that the core-valence spectrum in Fig. 2 is predominantly that of C$_3$O$_2$.

The chemically different carbon atoms in C$_3$O$_2$ give rise to individual core-valence spectra, where the core hole difference of 3.4 eV is expected to also be reflected in the spectrum. To test this hypothesis, two single ionization valence photoelectron spectra, shifted relative to each other according to the chemical shift of the two C 1s holes of C$_3$O$_2$ of 3.4 eV and folded in taking account of the relative intensity ratio of two C 1s photoelectron peaks of about 2:1, are included in black in the middle part of Fig. 2. Interestingly, the resulting spectrum based on this simple model, does not agree as well with the measured C 1s core-valence double ionization spectrum of C$_3$O$_2$ as is observed in some related cases^11^. This indicates a more complex description is needed, where the overlap of the core and valence orbital is included to calculate the singlet-triplet splitting for each core ionization site, as described and discussed below in the context of Fig. 4.

According to Gelius et al.^5^, C 1s ionization of C$_3$O$_2$ at high photon energy also gives rise to shake-up features about 8 eV and 10 eV above the edge, associated with the outer carbon atoms, while no corresponding shake-up contribution was reported for core ionization involving the central carbon atom. In our spectrum, shake-up contributions from the outer carbon atoms would be expected at 303 eV and 305 eV, where weak signs of intensity increase are indeed discernible in this region (cf. Fig. 2), but perhaps a bit too narrow to be associated with shake-up.

In Figs. 3 and 4 we show the results of the RASCF/RASPT2 calculations. The numerical method is validated by comparison of core and valence single ionization energies, shown in the Supplementary Table S1 and Fig. S1. The calculated core ionization energies for C 1s and O 1s are in excellent agreement with the experimental results from this work and previously published results from Gelius et al. (at MgK$_\alpha$ energy 1253.6 eV)^5^. The comparison of the calculated results with the conventional valence photoelectron spectrum of Rabalais et al.^4^(recorded at HeI excitation) shows excellent agreement as well, see Supplementary Table S1, and is also shown using our own valence photoelectron spectrum presented in Supplementary Fig. S1. Having thereby validated the technique, we moved on to the core-valence calculations, assuming a decoupling of the core and valence ionization events, thus obtaining a valence spectrum for each core ionized doublet potential of the molecule. As is clear from comparison with our experimental data in Fig. 4, the 5 main bands in the core-valence spectrum can be assigned assuming a four-fold representation of each state - singlet-triplet spin coupling and inner-outer carbon core. The main outer bands can be explained by molecular orbital (MO) theory, while smaller bands and inner lying bands in general have a more pronounced multi-configurational nature. From 325 eV ionization energy and onwards, the spectrum shows rather broad and faint features, which are probably due to effects of the breakdown of the MO picture, which become energetically possible in this high energy part. We also note accordingly that the OSRHF energies tend to overshoot in this part of the spectrum, see Table 1.

Comparing the core-valence spectra with the conventional valence photoelectron spectrum we notice some salient differences-apart from singlet-triplet splittings and, in the case of carbon core ionization, chemical shift splittings, one can observe that the spectrum is stretched out in the core-valence cases. In going from the features assigned to involving the 2$\pi _u$ to 6$\sigma _g$ orbitals, we see that the energy difference in the conventional valence photoelectron spectrum amounts to about 7 eV, while for the core-valence spectra it amounts to about 14 eV in the carbon case and about 20 eV in the oxygen case. These numbers are based on an estimate of the center of the split bands and are thereby quite rough. The wider spread of the core-valence spectra are expected because of the deeper potential generated by the additional hole. This can be observed by using other spectroscopy techniques, like XAS^12^, were the cationic forms give much wider spectra of discrete structures than the neutral spectra. It is also notable that the oxygen core-valence spectrum is wider than the carbon one, which may be understood by the fact that the core hole of the latter is somewhat more valence screened than the former. As noted above, towards higher ionization energies electron correlation (MO breakdown) effects kick in and become more prominent in the core-valence cases than in the valence photoelectron spectrum case. This owes also to the stronger potential that generates more possibilities for degeneracies, and also here we can compare with XAS spectra^12^. While it is not necessarily simple to compare numerical with experimental intensities because individual bands are overlapping, there are no drastic deviations between the coarse intensity profiles of the spectra which we compare here.

The energy separation of singlet and triplet core-valence states seems to depend strongly on the binding energy of the molecular orbital involved. Inspecting the oxygen spectrum, in Fig. 3, we see that the splitting is predicted to account for just a few tenths of an eV for the outermost $\sigma$ and $\pi$ states, while moving to deeper lying molecular orbitals the singlet-triplet splittings can amount to several eVs, cf. Table 1. The two carbon spectra, in Fig. 4, cover a smaller span in energy and also show a smaller variation in singlet-triplet splitting. One may interpret this as the 2s orbitals penetrating deeper into the core than 2p orbitals and that O 2s is deeper lying than C 2s, thereby generating stronger core-valence penetration based exchange interaction and singlet-triplet splitting in the spectra. The overall agreement between experimental and calculated spectra is better for the C1s edge in Fig. 4 compared to the O 1s edge in Fig. 3. The calculations are done within the sudden approximation limit, which assumes that the single-electron discrete-continuum couplings are the same for all transitions. This is generally a good approximation for high excitation energies, which is when the photon energy is high above the ionization potential. For the experimental spectrum above the O 1s edge, the photon energy at 618 eV is only about 10% above the core ionization energy, while the photon energy of 374 eV for the C 1s edge is 20% above the edge, which could possibly explain the better agreement for the C 1s edge. At the theory side it would be rewarding to develop a methodology that includes the continuum coupling elements explicitly, and interface that to the Dyson orbital method, perhaps using techniques like Stieltje’s imaging and B-splines that nowadays are used for normal UPS. This still would assume a decoupling of the valence and core electron ionization events, the conditions of which also calls for a closer scrutiny. Core-valence spectra for a selection of small molecules could then be used as a validation platform for such an expanded theory.

In Fig. 4, the calculation shows that the carbon core-valence spectra from the central and outer C atoms are chemically shifted relative to one another, where the onset of the outer carbon spectrum is found about 3 eV above the onset of the central carbon spectrum. This onset is assigned as a C1s $\pi$ transition in both cases. The central carbon core ionization energy is measured to be 3.4 eV higher than for the outer carbon thus conforming quite well with the shift in the core-valence spectral onsets. Such a large shift must primarily be due to charging, here that electron charge in the ground state is gathered at the center of the molecule, while charge at the ends is depleted, creating a stronger potential for the outer core electrons. The two carbon atoms also differ with respect to symmetry breaking and core hole localization, thus outer carbon ionization or core-valence excitation is expected to break molecular symmetry due to a dynamic pseudo Jahn-Teller interaction between symmetry adapted core hole states involving antisymmetric modes, while no such core hole localizing antisymmetric vibronic coupling is possible following the central carbon ionization. We have nevertheless assigned also the outer carbon core-valence spectra with u and g labels, according to how they correspond to the ground state orbitals, in order to facilitate comparisons. These symmetry breaking aspects were further discussed in our previous paper on allene^8^.

In the recent work on the core-valence spectrum of the allene molecule several computational methods were tested, including complete and restricted active space perturbation theory and open-shell restricted Hartree-Fock (OSRHF). The application of the former was concentrated to the lower part of the extended energy region covering core-valence states. As one can reach the basis set limit using OSRHF, see Ref.^8^, the results from this form of calculation represent a well-defined one-particle interpretation of the core-valence spectrum. Because it is described by the smallest possible expansion fulfilling spatial and spin symmetry, OSRHF excludes electron correlation effects. These effects are known to become progressively more important the deeper into the spectrum one reaches. This is already reflected in the conventional valence photoelectron spectrum, where both Koopmans’energies and OSRHF energies overshoot measured band energies. The interested reader is referred to Ref.^8^ for a more detailed analysis of this aspect. The capability of OSRHF to facilitate the assignment of the core-valence spectrum can also be judged by its performance for the regular valence and core photoelectron spectra, see Supplementary Table S1.

**Fig. 5:**
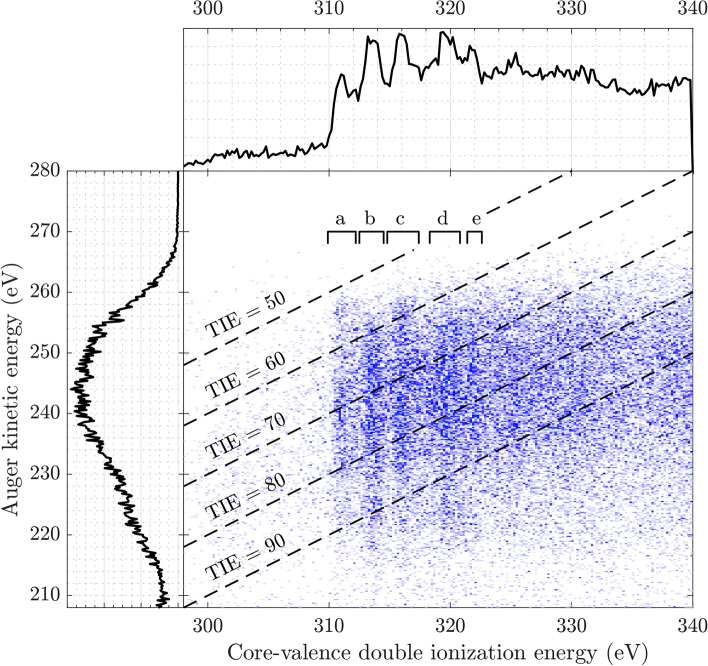
Coincidence map of core-valence double ionization energy and Auger electron kinetic energy, measured above the C 1s edge at a photon energy of 374 eV. The top spectrum shows the vertical sum of the map (same as the blue spectrum in Fig. 2), and the five peaks have been labelled by a-e. The left spectrum is the horizontal sum of the map. The dashed diagonal lines represent points of equal triple ionization energy (TIE).

To elucidate the assignments of the C1s core-valence features, we investigated the Auger decay upon core-valence double ionization and in comparison to the single core ionization case, respectively. For core-valence double ionization, the Auger spectra from selected energies in the core-valence double ionization spectrum are provisionally attributed to core ionization at the different C atoms. The coincidence map between the core-valence ionization energy and the Auger electron energy is shown in Fig. 5, where the five core-valence peaks have been labelled by a-e. We believe that the first and third peaks in the core-valence spectrum (a, c) involve the central carbon atom, while the second, fourth and possibly the fifth peaks relate predominantly to the outer carbon atoms (b, d, e). We see in this figure that core-valence ionization involving the central carbon atom is associated with higher kinetic energy Augers, while the higher energy hole states associated with the outer carbon atoms, give lower energy Auger electrons. If both Auger processes populated the same ground state of the final triply-charged ion the opposite energy ordering would be obtained. Thus the Auger process in this case seems to be selective in the choice of the orbital from which the valence electron is ejected.

To estimate the triple ionization energy for C$_3$O$_2$, the Auger electron energy is subtracted from the core-valence double ionization energy. In Fig. 5, the dashed lines represent points of constant triple ionization energies. We estimate the lowest triple ionization energy to about 52 ± 4 eV.

**Fig. 6:**
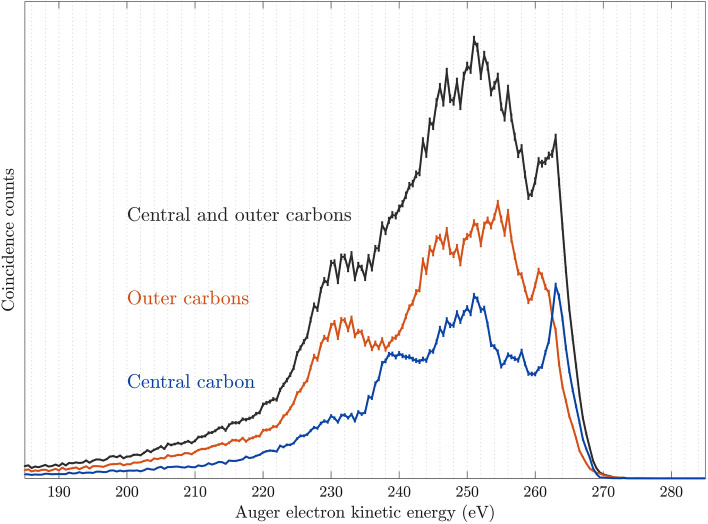
Auger electron spectra of C$_3$O$_2$ upon C 1s core ionization using a photon energy of 374 eV. In blue, the Auger electron spectrum related to a core vacancy on the central carbon atom, and in orange the corresponding spectrum involving the outer carbon atoms. The black spectrum is the sum of the blue and orange spectra.

We also note that the same effect is encountered in the Auger decay spectra from single core hole ionization, which are shown in Fig. 6. Here, the blue spectrum is extracted by selection on the 1s binding energy of the central carbon atom, and the orange spectrum is based on selection of the outer carbon 1s binding energy. Black reflects the sum of the two core-hole selected Auger spectra. As in the core-valence spectrum, the highest kinetic energy Auger electrons are associated with the central carbon atom. We note that Karlsson et al.^6^ reported the normal Auger electron spectra for C$_3$O$_2$ upon electron impact ionization at both the C 1s and the O 1s edges^6^. Taking the limited resolution of our high kinetic energy electrons into consideration, a comparison between the Auger spectra in their Fig. 1 to the total Auger spectrum we obtained for C 1s core ionization (black in Fig. 6) suggests the observation of very similar features. Comparison of the O 1s Auger spectra also shows good agreement of the overall structure.

For the C 1s vacancy, in Fig. 1 of Karlsson et al.^6^, the highest kinetic energy Auger electron is visible as a shoulder on the high energy side, attributed to the decay of the initial core vacancy on the outer carbon atoms. As one can see, this shoulder is not discernible in Fig. 6, possibly as a result of the difference in resolution, but from the core hole energy selected spectra included in the same figure, the highest energy Auger electrons are clearly associated with the central carbon. The peaks at highest kinetic energy for the blue and the orange spectra, 263 eV and 260.5 eV, are identified as the second and third peak listed in Ref.^6^, where they have energies of 261.7 eV and 259.4 eV. The peaks agree well in energy separation and are associated with the same core holes, central and outer, respectively, based on our spectra and the spectra of Karlsson et al.^6^.

A reason for the mismatching onsets of the Auger spectrum when derived from the outer and central core ionization sites, may be the operation of the effective one-center rule for Auger emission. The merits and limitations of this early proposed rule^13^ has been evaluated in numerous works studying molecular Auger^14^ processes. Thus from the molecular orbital picture shown in Fig. S2 of the Supplementary Materials we see that the outermost 2$\pi _\text{u}$ orbital has nodes at the outer carbon atoms while it has a maximal lobe at the central carbon. This indicates propensity for the outermost 2$\pi _\text{u}^{-2}$ doubly ionized states, namely $^1\Delta _\text{g}$ and $^1\Sigma _\text{g}$, to appear in the central rather than in the outer carbon Auger spectrum. This may explain the apparent shift of the onsets of the doubly ionized states when derived from these two spectra, as described in Karlsson et al.^6^. Further numerical evaluation of the core-valence Auger spectra has to await a further study.

## Conclusions

We have measured the core-valence double ionization spectra of C$_3$O$_2$ , and also the Auger spectra of the same molecule with full specificity in choice of the atom whose 1s core electrons are initially ionized. The core-valence spectrum from 1s electron ejection at the C 1s edges is quite well matched by RASSCF/RASPT2 calculations, while the spectrum from O 1s ionization is fitted less well. Auger electron spectra at the central and outer C 1s edges show marked selectivity in the final doubly-charged states reached. A similar but more complex selectivity is observed in Auger spectra from the core-valence states, and will be the subject of further investigations.

## Methods

### Multi-electron correlation spectroscopy and light sources

Multi-electron coincidence experiments were carried out at the synchrotron radiation facility PETRA III at DESY in Hamburg, and in our laboratory at the University of Gothenburg using a pulsed He gas discharge lamp. The magnetic bottle time-of-flight spectrometer used for the present work is a more compact version of the original instrument, described by Eland et al. in Ref.^15^, while otherwise being very similar in design and working principle. In short, the setup allows for efficient measurement of electron coincidence spectra, with a collection-detection efficiency of 50-60 % and a nominal resolving power of *E*/$\Delta E$ = 50. The gaseous sample is let into the light-matter interaction region by a hollow needle, providing an effusive jet of gas. Upon ionization of the sample by the intersecting light, electrons are confined by a divergent magnetic field provided by a conical magnet of 1 T, entering a 2 m long flight tube. This magnetic field couples to an about 1 mT weak homogeneous field created by a solenoid surrounding the flight tube. At the end of the flight tube a multi-channel plate detector is used for recording the arrival flight times of the electrons. The flight times of the electrons are converted to kinetic energies, where the time-to-energy conversion was based on known Auger and photoelectron spectra measured for a series of standard calibration gases such as Kr and Ar.

At PETRA III, our setup was connected to beam line P04^16^. Photon energies of 310 eV, 374 eV and 618 eV were used for the high statistics measurements of the present experiments, where the lowest energy was used for monitoring possible contaminants, and the higher photon energies to measure the here presented core-valence ionization spectra. At PETRA III, the light’s inter-pulse spacing, when operated in 40-bunch mode, is 192 ns, which is too short for our coincidence detection system to work unambiguously. This spacing was increased to about 10 $\mathrm {\mu }$s using a new, much refined version of the mechanical chopper reported in Ref.^17^, fully synchronized to the radio frequency signal of the storage ring, building on a recent technological development done at the MAX-IV laboratory in Lund.

In our laboratory at the University of Gothenburg, a pulsed helium discharge lamp provided the ionizing radiation pulses. It operates at a repetition rate of 4 kHz, corresponding to a 250 $\mathrm {\mu }$s inter-pulse spacing. From the helium lamp, photon energies of 21.2 and 40.8 eV were selected by means of a toroidal monochromator built in our laboratory and used for obtaining valence single ionization electron spectra.

### Synthesis of target sample

Carbon suboxide was synthetized by dehydration of malonic acid with P$_2$O$_5$ following the recipe known in the literature^18,19^. The reaction

$$\begin{aligned} {{2 \text{C}_{3}\text{H}_{4}\text{O}_{4} \mathop {\longrightarrow }\limits ^{145^\circ \text{C}}_{ \text{P}_{2} \text{O}_{5}} \text{C}_{3}\text{O}_{2} + \text{CO}_{2} + \text{CH}_{3}\text{COOH} + 2 \text{H}_{2}\text{O}}} \end{aligned}$$

was set to occur at 145$^\circ$C, where the major gaseous product is expected to be CO$_2$, with C$_3$O$_2$ and acetic acid vapour (CH$_3$COOH) as minor products.

**Fig. 7:**
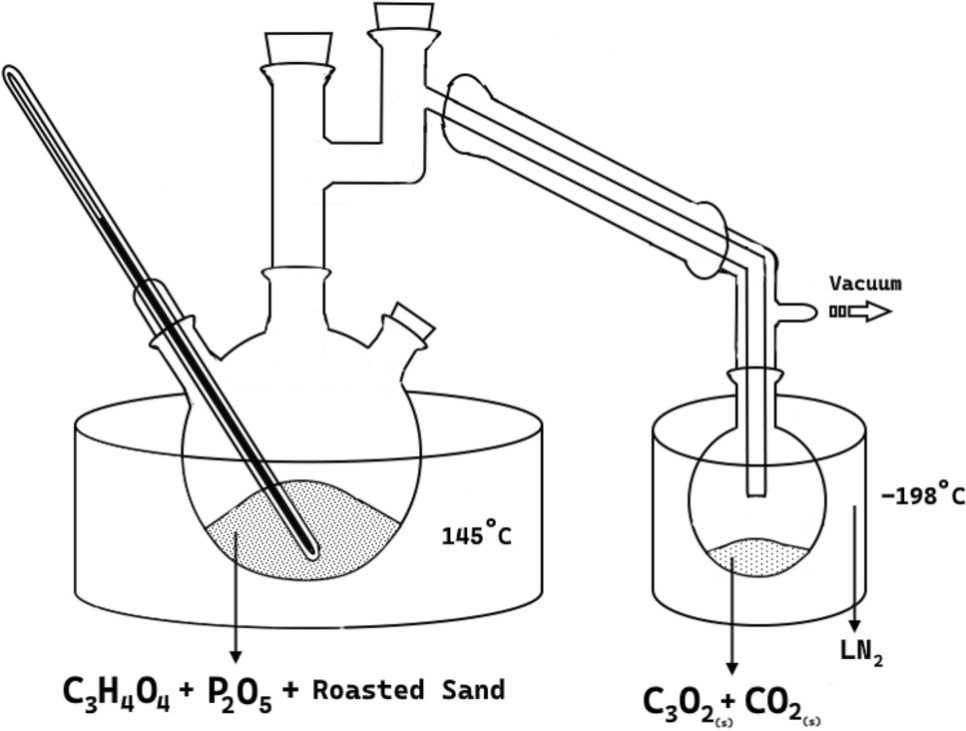
Illustration of the experimental setup for the synthesis of C$_3$O$_2$.

The synthesis setup illustrated in Fig. 7 consisted of a three-necked flask, fitted with a thermometer and either a Liebig condenser without flowing water or a simple bridge, attached to a receiving flask placed in a dewar filled with liquid nitrogen. The entire reaction took place under vacuum (about 10$^{-2}$ mbar). 10 to 12 g of malonic acid were finely ground and dried in a desiccator for a few days, then mixed with 50 g of P$_2$O$_5$ and 20 g of roasted sand before being added to the main flask. The latter was previously cleaned and set under vacuum to remove any trace of moisture.

The flask was placed on a heating mantle to control the temperature. Although externally 145$^\circ$C was reached, heat losses and the conductivity of the flask meant that the internal temperature reached a peak of about 130 $^\circ$C. During the course of two hours, a white solid condensed in the receiving flask. At the end of the reaction, the receiving flask was removed from the system and the product was left to thaw under the fume hood because of the lacrimatory effect that C$_3$O$_2$ possesses, and was transferred into a test tube with a KF junction when it became liquid (C$_3$O$_{2mp}$ = -110$^\circ$C). The sample was then purified by being placed in an ethanol/LN$_2$ bath at -78$^\circ$C and pumped for several cycles, to remove all CO$_2$. Only small amounts of acetic acid were detected experimentally. At the end, 1 to 1.5 ml of carbon suboxide had been collected, corresponding to a yield of about 10 $\%$, consistent with the rate previously reported in the literature^18,19^. Because of its highly unstable nature, each sample batch was available for experimental runs for no longer than two days at room temperature, as the polymerization reaction takes place despite the purification.

### Computational details

The equilibrium geometry of the carbon suboxide molecule was obtained using density functional theory (DFT), at the DFT/B3LYP/aug-cc-pVTZ level. All electronic transitions have been described keeping the geometry fixed. The singly- and doubly-ionized states were characterized with multi-configuration wave function methods. Particularly, we employed the restricted active space self-consistent field (RASSCF) and the second order of perturbation (RASPT2) techniques, as implemented in the OpenMolcas package^20^. To keep track of the different core sites, the core orbitals were localized according to a Pipek-Mezey procedure^21^. The localization required the calculations to be performed in the $C_{2v}$ point group of symmetry, so the orbitals (states) belong to the $a_1$ (A$_1$), $a_2$ (A$_2$), $b_1$ (B$_1$) and $b_2$ (B$_2$) symmetry components. Two different active spaces have been explored. In the first, the ionized core orbital was included in RAS1 and all valence orbitals were included in RAS2. When restricting the number of holes in RAS1 and RAS2, all singly-ionized and core-valence doubly-ionized states were virtually described by a single determinant. For that reason we refer to this active space as being equivalent to the open-shell restricted Hartree-Fock (OSRHF), although we allow the orbitals to be state-average optimized. The second active space includes 10 extra orbitals in RAS3 - 4 $a_1$, 2 $a_2$, 2 $b_1$ and 2 $b_2$ virtual orbitals - allowing a maximum of two electrons in this subspace. The energy of the principal final states linked to the conventional valence photoelectron and all core-valence spectra were obtained using both active spaces, and the results are shown in Table 1 and Supplementary Table S1.

The conventional valence photoelectron and core-valence spectra were simulated considering the second active space. For the conventional valence photoelectron spectrum, the active space totalizes 17314 configuration space functions (CSFs) for the doublet final states belonging to the A$_1$ symmetry and 17138 CSFs for the $B_1$ symmetry states. In contrast, for the core-valence spectra the size of the configuration space was different for singlet and triplet final sates. For the singlet states the number of CSFs are naturally the same as the ones for the conventional valence photoelectron spectrum. For the triplet states the active space totalizes 29322 and 29241 CSFs for the A$_1$ and B$_1$ symmetries, respectively. These numbers are the same for all core-hole sites. The intensities have been addressed at the sudden approximation level^20,22^, which in turn is based on the evaluation of the Dyson orbitals in conformity with the RAS State Interaction suit^23–27^. For the core-valence doubly-ionized states we approximate the transition amplitudes by the products of the Dyson amplitudes for the core single-ionization - starting from the neutral species - and the valence single-ionization - starting from the core-hole state. In other words, we addressed the double ionization as consisting of two-step processes described by two sequential sudden approximations. In the calculated core-valence spectra, single state energies have been artificially broadened by Gaussian profiles of 0.5 and 0.7 eV FWHM for the C 1s and O 1s case, respectively, to match the experimental energy resolution over the spectra. The intensities were based on the product of the Dyson transition probabilities, resulting in an overall aspect ratio of 3:1, thus reflecting well the expected weighting for triplets and singlets.

**Table 1:** Comparison of calculated C 1s and O 1s core-valence double ionization energies for C$_3$O$_2$, in eV. The spin multiplicity is denoted by T (triplet) or S (singlet), the relative intensities are given in the fourth column, and the rightmost column show the valence molecular orbital for the main peaks in Figs. 3 and 4.

State	OSRHF	RASPT2	Int.	V. orb.
C$_\text{C}$($\pi$) T	311.49	311.35	0.85	2$\pi _\text{u}$
C$_\text{C}$($\pi$) S	312.33	312.35	0.41	2$\pi _\text{u}$
C$_\text{O}$($\pi$) S	315.05	314.21	0.43	2$\pi _\text{u}$
C$_\text{C}$($\pi$) S	315.56	314.28	0.36	1$\pi _\text{g}$
C$_\text{O}$($\pi$) T	314.94	314.37	0.87	2$\pi _\text{u}$
C$_\text{C}$($\pi$) T	315.56	314.47	0.72	1$\pi _\text{g}$
C$_\text{C}$($\pi$) T	317.26	315.85	0.75	1$\pi _\text{u}$
C$_\text{C}$($\sigma$) T	318.62	316.39	0.76	5$\sigma _\text{u}$
C$_\text{C}$($\sigma$) S	318.67	316.41	0.37	5$\sigma _\text{u}$
C$_\text{C}$($\sigma$) T	318.81	316.51	0.75	
C$_\text{C}$($\sigma$) S	318.88	316.53	0.37	
C$_\text{C}$($\pi$) S	317.78	316.69	0.37	1$\pi _\text{u}$
C$_\text{O}$($\pi$) S	321.32	318.49	0.36	1$\pi _\text{g}$
C$_\text{O}$($\pi$) T	321.30	318.80	0.73	1$\pi _\text{g}$
C$_\text{O}$($\pi$) T	323.24	319.81	0.75	1$\pi _\text{u}$
C$_\text{O}$($\sigma$) S	324.13	320.21	0.37	5$\sigma _\text{u}$
C$_\text{O}$($\sigma$) T	324.12	320.36	0.75	5$\sigma _\text{u}$
C$_\text{O}$($\pi$) S	324.11	321.51	0.38	1$\pi _\text{u}$
C$_\text{O}$($\sigma$) T	325.27	321.62	0.78	6$\sigma _\text{g}$
C$_\text{O}$($\sigma$) S	326.10	322.43	0.23	6$\sigma _\text{g}$
C$_\text{C}$($\sigma$) T	326.42	324.47	0.32	6$\sigma _\text{g}$
C$_\text{C}$($\sigma$) S	327.37	325.38	0.39	6$\sigma _\text{g}$
C$_\text{O}$($\sigma$) S	332.20	327.81	0.19	
C$_\text{O}$($\sigma$) T	331.45	327.83	0.73	
C$_\text{C}$($\sigma$) T	331.35	327.89	0.30	
C$_\text{O}$($\sigma$) S	337.40	329.07	0.33	
C$_\text{C}$($\sigma$) T	338.77	329.17	0.14	
C$_\text{C}$($\sigma$) S	334.36	329.77	0.18	
C$_\text{O}$($\sigma$) T	335.88	331.73	0.05	
C$_\text{C}$($\sigma$) S	338.77	333.26	$<10^{-2}$	
C$_\text{C}$($\sigma$) T	338.83	334.30	0.18	
C$_\text{C}$($\sigma$) S	338.89	334.44	0.13	
C$_\text{O}$($\sigma$) T	344.18	336.09	0.08	
C$_\text{O}$($\sigma$) T	345.70	337.38	0.05	
C$_\text{O}$($\sigma$) S	344.18	339.05	0.08	
C$_\text{O}$($\sigma$) S	347.36	339.77	$<10^{-2}$	
O($\pi$) T	558.44	559.11	0.87	2$\pi _\text{u}$
O($\pi$) S	558.64	559.30	0.43	2$\pi _\text{u}$
O($\pi$) S	564.13	562.79	0.37	1$\pi _\text{g}$
O($\pi$) T	564.14	562.90	0.73	1$\pi _\text{g}$
O($\sigma$) S	566.55	564.33	0.37	5$\sigma _\text{u}$
O($\sigma$) T	566.56	564.52	0.75	5$\sigma _\text{u}$
O($\pi$) T	568.16	566.79	0.76	1$\pi _\text{u}$
O($\sigma$) T	570.65	568.86	0.78	6$\sigma _\text{g}$
O($\pi$) S	570.36	569.51	0.38	1$\pi _\text{u}$
O($\sigma$) S	571.83	570.19	0.19	6$\sigma _\text{g}$
O($\sigma$) T	572.89	571.02	0.31	
O($\sigma$) S	574.35	572.59	0.33	
O($\sigma$) S	577.31	573.81	0.31	
O($\sigma$) T	577.18	574.01	0.73	
O($\sigma$) T	586.63	579.36	0.15	
O($\sigma$) S	586.61	580.09	0.04	
O($\sigma$) S	595.47	582.98	0.04	
O($\sigma$) T	590.83	583.73	0.10	

dummy

## Supplementary Information

Supplementary Information.

## Data Availability

The data sets generated during and/or analysed during the current study are available from the corresponding authors on reasonable request.
