# Supplementary material for: Core-valence double ionization of carbon suboxide
Source: Sci Rep. 2025 May 6;15:15765. doi: 10.1038/s41598-025-01057-4 (PMC12056205; doi:10.1038/s41598-025-01057-4)
Supplement: Supplementary file 1 — Supplementary Information. [file 41598_2025_1057_MOESM1_ESM.pdf]

Supplementary Materials: Core-valence double ionization of carbon suboxide

Emelie Olsson[1], Lucas M. Cornetta[2], Veronica Daver Ideböhn[1], Måns Wallner[1], Marco Parriani[1,3], Richard J. Squibb[1], Gunnar Öhrwall[4], Stefano Falcinelli[3], Leif Karlsson[5], John H. D. Eland[6], Hans Ågren[5,7], and Raimund Feifel[1,*]

[1]University of Gothenburg, Department of Physics, Origovägen 6B, 412 58 Gothenburg, Sweden
[2]Instituto de Física da Universidade de São Paulo, R. do Matão, 1371, 05508-090, São Paulo, Brazil.
[3]University of Perugia, Department of Civil and Environmental Engineering, Via G. Duranti 93, 06125 Perugia, Italy
[4]MAX IV Laboratory, Lund University, Box 118, 221 00 Lund, Sweden
[5]Uppsala University, Department of Physics and Astronomy, Box 516, SE-751 20 Uppsala, Sweden
[6]Oxford University, Department of Chemistry, Physical and Theoretical Chemistry Laboratory, South Parks Road, Oxford OX1 3QZ, United Kingdom
[7]Faculty of Chemistry, Wroclaw University of Science and Technology, Wyb. Wyspianskiego 27, PL-50370 Wroclaw, Poland
[*]corresponding author: raimund.feifel@physics.gu.se

Table S1. Comparison of single ionization energies of C_3O_2
Energies calculated with OSRHF and Koopmans energies for UPS (eV). Comparison to our experimental values, peak energies from Gelius et al.[1] and adiabatic ionization energies from Rabalais et al.[2].
[*]adiabatic ionization energies

Spec.	State	OSRHF	Koop.	RASPT2	Exp. (this work)	Gelius et al.	Rabalais et al.[*]
UPS	$2\pi_u$	10.52	11.15	10.86	10.9 ± 0.5	10.8 ± 0.2[1]	10.61[2]
	$1\pi_g$	16.74	17.40	15.21	14.9 ± 0.5	15.0 ± 0.2[1]	14.55[2]
	$1\pi_u$	17.67	18.26	16.07	15.9 ± 0.5	16.0 ± 0.2[1]	15.75[2]
	$5\sigma_u$	19.60	20.43	16.84	17.2 ± 0.5	17.3 ± 0.1[1]	16.98[2]
	$6\sigma_g$	19.97	20.76	17.07		17.5 ± 0.1[1]	17.26[2]
	$4\sigma_u$	25.02	25.69	22.09	21.7 ± 0.5	21.9 ± 0.2[1]	
	$5\sigma_g$	29.48	30.32	26.16	25.3 ± 0.5	25.6 ± 0.1[1]	
	$3\sigma_u$	39.71	40.73			35.5 ± 0.1[1]	
	$4\sigma_g$	39.75	40.76			35.5 ± 0.1[1]	
XPS	C_C 1s	291.61	306.53	291.48	291.5 ± 1	291.4 ± 0.1[1]	
	C_O 1s	296.88	311.28	295.43	294.9 ± 1	294.9 ± 0.1[1]	
	$I(C_O/C_C)$				> 2	1.8[1]	
	O 1s	539.90	562.38	539.69	539.7 ± 1	539.7 ± 0.1[1]	

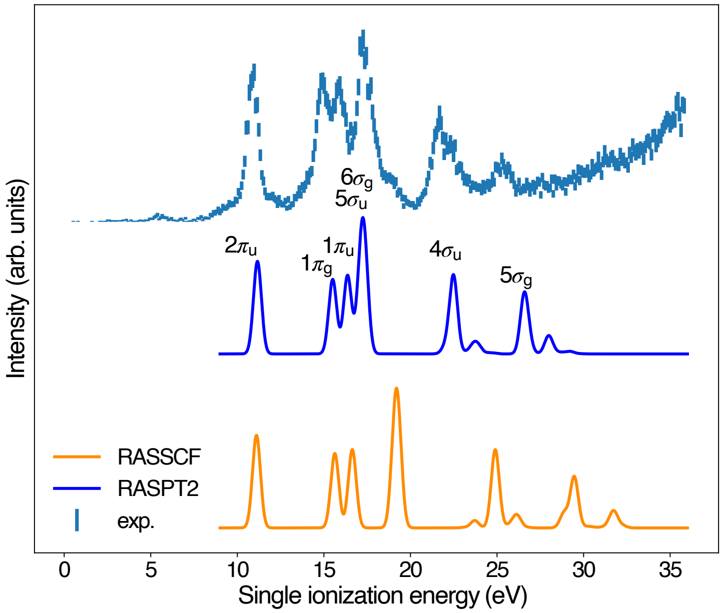

Figure S1. Comparison of the experimental single ionization electron spectrum taken at 40.81 eV (top) and the computed spectra from RASPT2 (middle) and RASSCF (bottom).

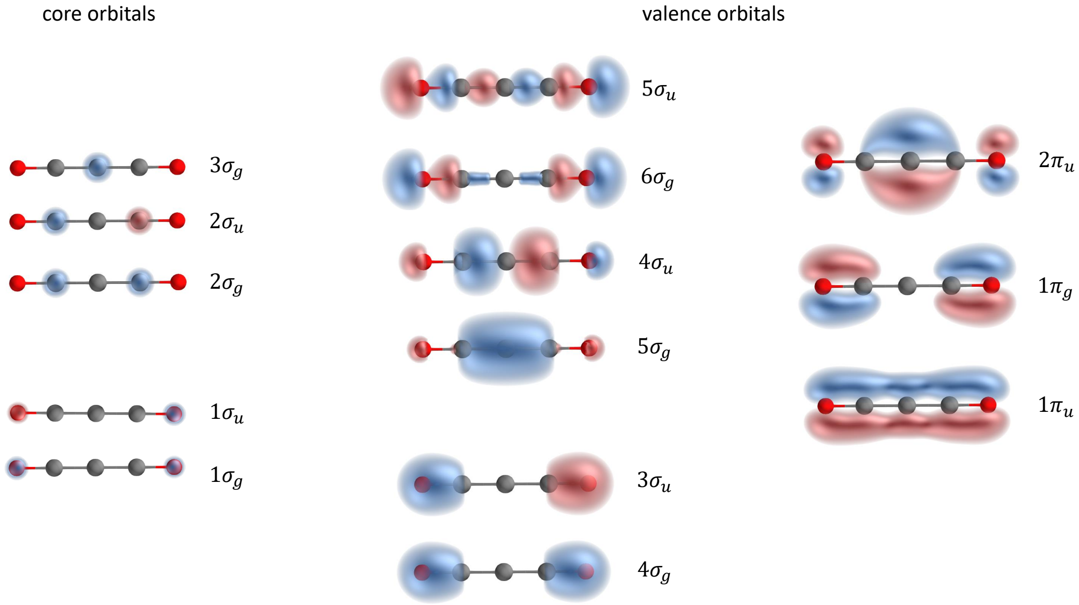

Figure S2. Molecular orbitals of carbon suboxide.

References

1. Gelius, U., Allan, C., Allison, D., Siegbahn, H. & Siegbahn, K. The electronic structure of carbon suboxide from ESCA and AB initio calculations. *Chem. Phys. Lett.* **11**, 224–228, DOI: https://doi.org/10.1016/0009-2614(71)80364-0 (1971).

2. Rabalais, J. W. *et al.* The high-resolution electron spectrum of carbon suboxide. *Electron Spectrosc.* (1972).
